# Intestinal Lymphangiectasia as a Rare Myeloma-Defining Event

**DOI:** 10.7759/cureus.99226

**Published:** 2025-12-14

**Authors:** Lateefa Rashed Daraj, Shruti Prem Sudha, Mahera Roohi

**Affiliations:** 1 Department of Hematology, Bahrain Oncology Center, Manama, BHR; 2 Department of Pathology, Blood Bank and Laboratory Medicine, King Hamad University Hospital, Manama, BHR

**Keywords:** malabsorption, myeloma, paraprotein, protein-losing enteropathy, secondary lymphangiectasia

## Abstract

Multiple myeloma is a clonal plasma cell disorder with a pre-malignant phase in the form of monoclonal gammopathy of uncertain significance (MGUS) and smoldering myeloma. Progression from MGUS or smoldering myeloma to overt myeloma is usually associated with myeloma-defining events described in the acronym CRAB (hypercalcemia, renal insufficiency, anemia, and bone lesions). Herein we present a patient who presented with protein-losing enteropathy as the initial myeloma-defining event, secondary to intestinal lymphangiectasia caused by the immunoglobulin deposition and obstruction of intestinal lymphatics. After initiating treatment for myeloma, her symptoms rapidly resolved. We report this case to describe a rare myeloma-defining event caused by the raised paraprotein. This case illustrates the protean clinical manifestations in myeloma, and the importance of not restricting one’s thinking to the traditional CRAB criteria, since delay in diagnosis of myeloma may result in end organ damage, complications, and increased morbidity.

## Introduction

Multiple myeloma is a clonal plasma cell disorder, characterised by the accumulation of an abnormal paraprotein in the blood, composed of immunoglobulins secreted by the clonal plasma cells. Myeloma is consistently preceded by a pre-malignant phase termed monoclonal gammopathy of undetermined significance (MGUS), and an intermediate stage called smoldering myeloma, where there is no end organ damage [1,2]. Overt myeloma is diagnosed by the presence of end organ damage, called myeloma-defining events as described in the acronym CRAB: hypercalcemia, renal insufficiency, anemia, and lytic bone lesions [3,4]. CRAB signs or symptoms arise from the infiltration of plasma cells into the bone or other organs, or due to renal tubular damage from immunoglobulin light deposition. While the progression from MGUS or smoldering myeloma to overt myeloma is usually subacute, a small percentage of patients present acutely, with symptoms requiring rapid attention and intervention (eg, spinal cord compression from plasma cell deposits, acute renal failure, hypercalcemia) [5]. Complications caused by the excess paraprotein such as hyperviscosity or lymphatic obstruction can also present acutely. Intestinal lymphangiectasia is one such rare complication described due to the excess paraprotein [6-8]. This is a rare condition characterized by obstruction of the intra-mucosal lymphatics of the small bowel, causing them to leak lymph fluid containing proteins back into the intestine. This leads to malabsorption of fats and proteins, resulting in symptoms like edema, fatigue, diarrhea, and abdominal pain. Primary intestinal lymphangiectasia, also called idiopathic, occurs congenitally in the absence of causative factors. Secondary intestinal lymphangiectasia is caused by other conditions that obstruct lymphatic flow, such as tumors, infections, inflammation, or heart diseases. Diagnosis is confirmed through a biopsy, and management typically involves a low-fat, high-protein diet and correction of the underlying disorder in secondary causes. 

Here we report a patient with profuse watery diarrhea and weight loss as the initial myeloma-defining event, caused by secondary intestinal lymphangiectasia due to obstruction of intestinal lymphatics by the myeloma paraprotein. Manifestations of non-CRAB end organ damage (eg, hyperviscosity, damage due to the raised paraprotein, recurrent infections, peripheral neuropathy) are nonspecific and not diagnostic of myeloma. We present this case to highlight an uncommon myeloma-defining event driven by elevated paraprotein levels. Major delays in diagnosis have been associated with a negative impact on the disease course in myeloma. Consequently, clinicians should always take into account the rare presentations of myeloma to avoid delays in diagnosis and treatment, which could result in increased morbidity and mortality.

## Case presentation

A 62-year-old woman with a history of treated breast cancer was under follow-up for smoldering myeloma (IgG kappa subtype) for the last three years. She had iron deficiency anemia that was corrected, and was on follow-up without the appearance of any other traditional myeloma-defining event in the form of hypercalcemia, renal impairment or lytic bone lesions. She was undergoing follow-up with myeloma labs every three months and the last laboratory evaluation (Table 1) was consistent with smoldering myeloma with hemoglobin of 11 g/dl, M band of 2.5g/dl, IgG of 27g/l, and kappa/lambda (K/L) ratio of 2.4, and bone marrow aspiration showed 50% plasma cells. As per the last evaluation she did not fulfil the CRAB criteria for progression to overt myeloma, and the K/L ratio was less than 100, and bone marrow plasma cell percentage was less than 60%, which did not fulfil SLiM (≥60% marrow plasma cells, ≥100 free Light chain ratio, >1 focal lesion on MRI)-CRAB criteria for evolution to myeloma.

**Table 1:** Temporal course of hematological and biochemical parameters over the three-year period

Parameter	Initial Labs	Labs after three years at the time of diarrhea	Reference Range
Hemoglobin (Hb)	11.0 g/dl	8.7 g/dl	12-16 g/dl
Calcium	1.3 mmol/L	1.87 mmol/L	2.12-2.62 mmol/L
Creatinine	55.4 umol/L	40.8 umol/L	49-90 umol/L
Total protein	78.7 g/L	86.9 g/L	60-83 g/L
Albumin	35.8 g/L	33.0 g/L	38 -50 g/L
Globulin	42.9 g/L	53.9 g/L	20-35 g/L
Immunoglobulin G (IgG)	27.0g/L	32.0g/L	6.0 to 16.0 0 g/L
Kappa/lambda ratio (K/L)	2.40	1.33	0.31-1.56
M band on electrophoresis	2.5g/dl	3.2g/dl	Not detectable
Bone marrow aspirate	50% clonal plasma cells	76% clonal plasma cells	No clonal plasma cells

**Figure 1:**
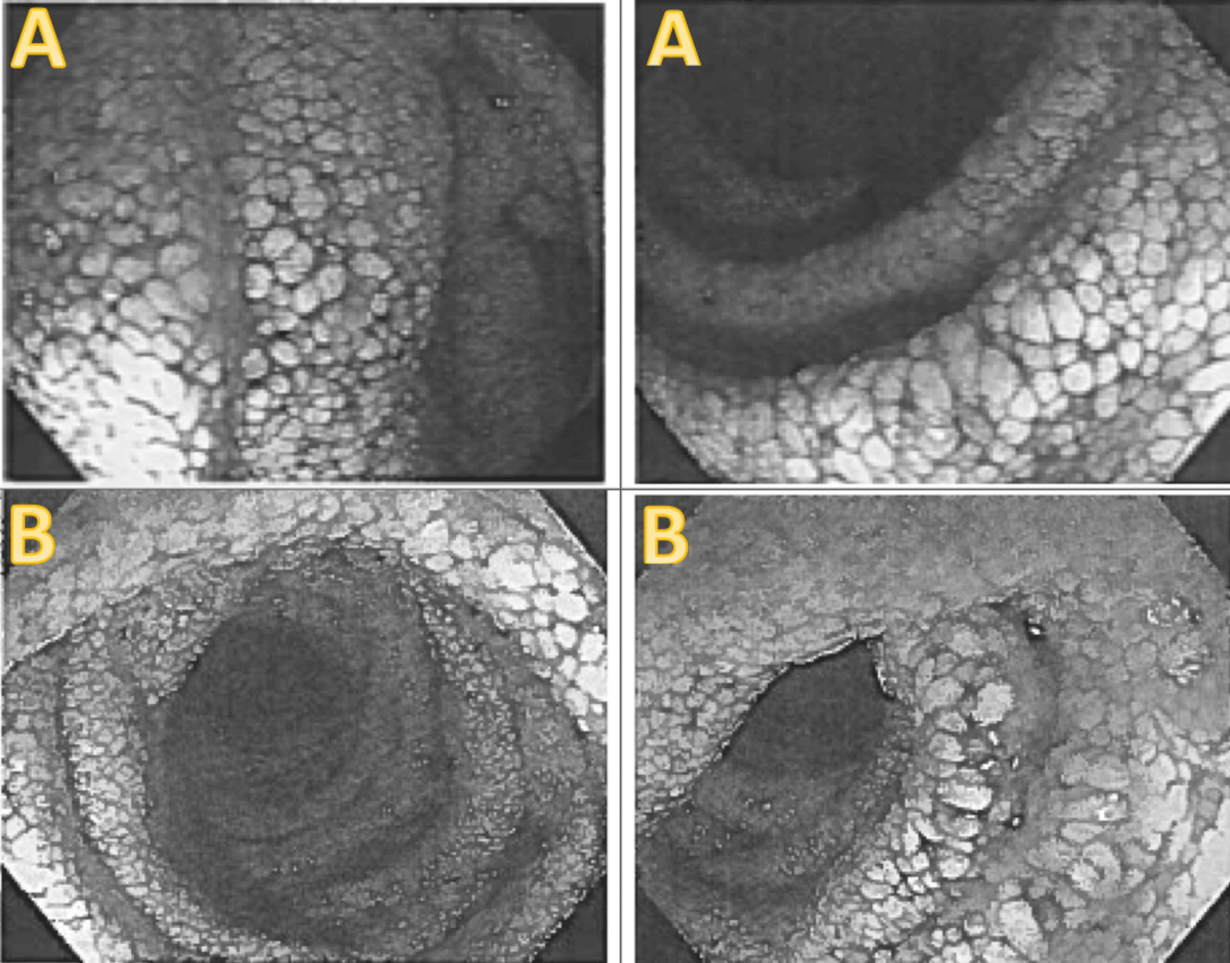
Diffusely edematous villi of (A) duodenum and (B) jejunum due to lymphatic obstruction

**Figure 2:**
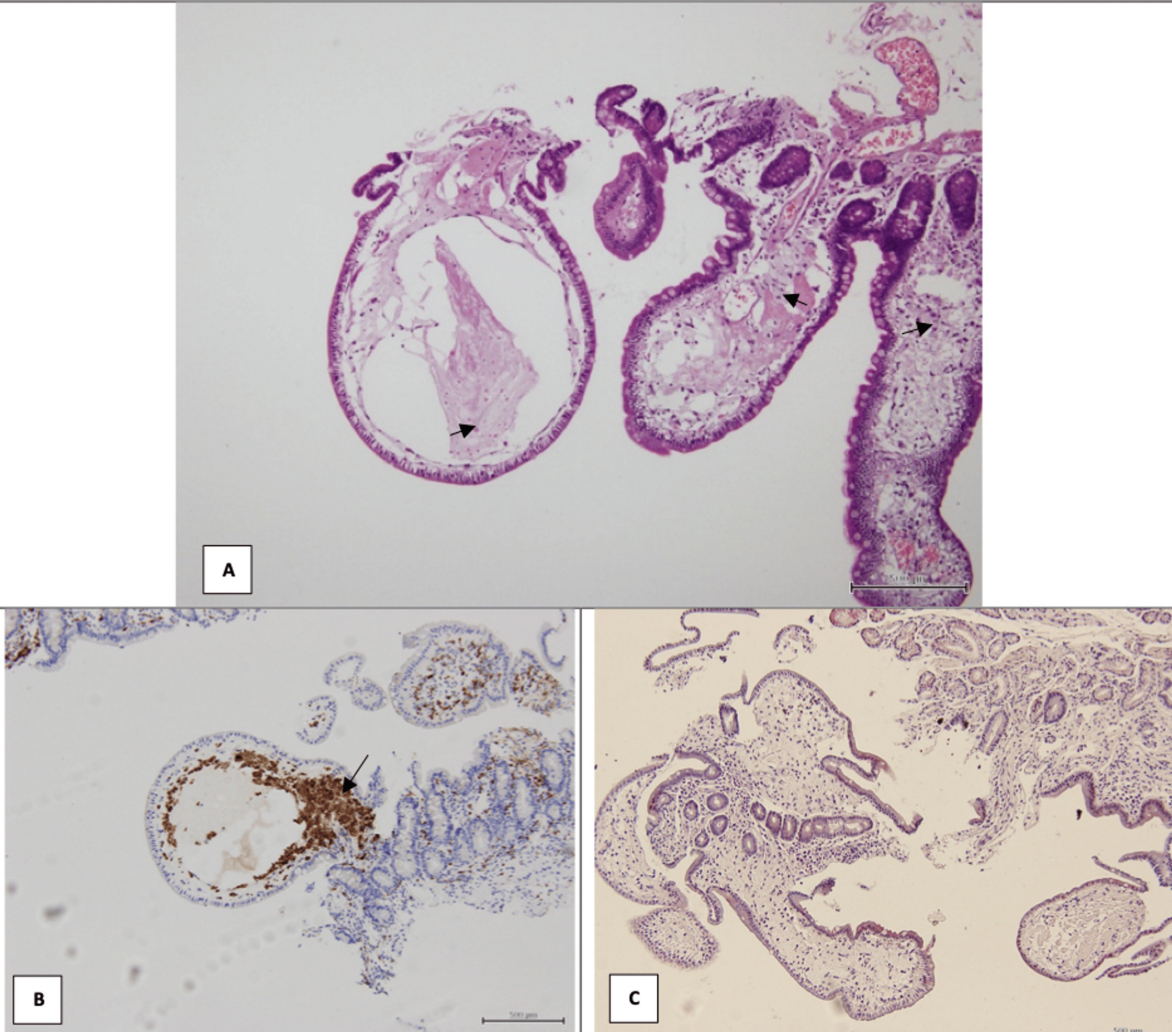
Biopsies from the (A) fourth part of the duodenum and (C) jejunum, showed markedly distended and edematous villi consistent with lymphangiectasia, and (B) CD68 positivity highlighting foamy macrophages within the intestinal villi.

During the third year of follow-up, she underwent evaluation in an overseas hospital for profuse watery diarrhea, abdominal pain, and weight loss of 13 kg. She was initially presumed to have Crohn’s disease and received mesalazine and mebeverine without improvement in her symptoms. She underwent a CT scan of the abdomen which showed diffuse small bowel thickening involving the second part of the duodenum, jejunum, and ileum. Upper GI endoscopy revealed whitish villi in the duodenum and colonoscopy showed diverticulosis. The biopsies from the colon, terminal ileum, and second part of the duodenum were normal. She then underwent push enteroscopy which showed diffuse grossly edematous whitish villi in other parts of the duodenum and jejunum (Figure 1). Biopsies from the fourth part of the duodenum and jejunum showed markedly distended and edematous villi containing foamy macrophages positive for CD68. The appearance was consistent with lymphangiectasia (Figure 2).

She was started on octreotide injections at the overseas hospital showing partial improvement in the diarrhea. She subsequently returned to our clinic and the intestinal pathological findings raised the suspicion of secondary telangiectasia due to obstruction of intestinal lymphatics by the myeloma protein, hence she underwent reassessment for myeloma status (Table 1). Hb at this juncture was 8.7g/dL, however improved to 11 g/dLafter correction of iron deficiency anemiaand calcium and creatinine were normal and there were no lytic lesions on skeletal imaging. Myeloma profile at this juncture showed IgG of 32g/l, K/L ratio was 1.3, and M band of 3.2g/dl, which was not markedly increased compared to the previous report. At this point she still did not fulfil the CRAB criteria, however repeat bone marrow assessment was done which showed 76% plasma cells, suggestive of progression to overt myeloma based on SLiM-CRAB criteria where bone marrow clonal plasma cell percentage more than 60% is considered diagnostic of overt myeloma even in the absence of CRAB.

The secondary lymphangiectasia and resultant malabsorption symptoms were considered to be a myeloma-defining event necessitating initiation of myeloma-directed therapy. Treatment for myeloma was commenced with triple drug regimen (daratumumab, lenalidomide, and dexamethasone). This resulted in rapid improvement in diarrhea, and weight gain. Myeloma profile and bone marrow evaluation were repeated after four cycles of this regimen and were consistent with complete remission. She was offered stem cell collection followed by high-dose chemotherapy and autologous stem cell transplantation as consolidation for the myeloma, but declined the same. She completed six cycles of daratumumab, lenalidomide, and dexamethasone, and is currently on long-term maintenance with lenalidomide without evidence of recurrence of myeloma.

## Discussion

According to the International Myeloma Working Group criteria, a diagnosis of overt myeloma is made when the bone marrow is infiltrated by >10% clonal plasma cells, and there is the presence of one of the myeloma-defining events described in the CRAB criteria [3-5]. Our patient was on regular follow-up for smoldering myeloma and did not fulfill traditional myeloma-defining criteria. She presented with gastrointestinal symptoms from protein-losing enteropathy and malabsorption secondary to secondary lymphangiectasia, presumably due to blockage of intestinal lymphatics by the myeloma paraprotein. This prompted the initiation of myeloma treatment which was associated with rapid resolution of symptoms.

Myeloma can cause gastro-intestinal pathology by several mechanisms including direct infiltration, amyloidosis, or local plasmacytoma [6-11]. Secondary lymphangiectasia due to the raised paraprotein has been reported rarely in literature [6-8]. Bajwa et al. [6] and Bhat et al. [7] have reported in patients with smoldering myeloma and MGUS respectively, small intestinal lymphangiectasia causing steatorrhea and weight loss and necessitating myeloma-directed therapy. The mechanism of lymphangiectasia in these cases was presumed to be immunoglobulin deposition causing increased interstitial viscosity and lymphatic obstruction which led to protein-losing enteropathy and malabsorption. A different mechanism of lymphangiectasia was reported by Salguero et al. [8] in a patient where myelomatous infiltration of the mesenteric lymph nodes was purported to be the cause of secondary intestinal lymphangiectasia.

The differential diagnosis of secondary lymphangiectasia is broad including lymphoma, celiac disease, Whipple disease, Crohn’s disease, and myeloma among other causes [12-14]. Typical clinical features are consistent with protein-energy malnutrition; in addition, impaired cell-mediated immunity, hypogammaglobulinemia, and lymphocytopenia may be seen. Typical features on endoscopy include dilated lymphatic lacteals, which appear as white-tipped villi. The standard for diagnosis of intestinal lymphangiectasia is a small-intestine biopsy revealing dilated lacteals with villus blunting. Octreotide has been shown to have success in symptom control in such cases [15], but definitive management of secondary lymphangiectasia should be tailored to the cause.

Our case has unique features in that the patient did not have the traditional CRAB criteria for myeloma but had a very rare myeloma-defining event, namely protein-losing enteropathy and malabsorption due to obstruction of intestinal lymphatics by the abnormal paraprotein. A similar presentation has been reported in only two cases in literature before. From the perspective of a gastroenterology specialist, it may not be intuitive to consider myeloma as a cause for chronic diarrhea, and indeed initially our patient was presumed to have Crohn’s disease, and received treatment for the same without clinical improvement. Biopsies from the stomach and the first part of the duodenum were non-contributory, and the characteristic findings of intestinal lymphangiectasia were identified on push enteroscopy and biopsy from the fourth part of the duodenum. The pathological findings were congruent with her clinical symptoms and suggested the possible mechanism of obstruction of the intestinal lymphatics by the raised paraprotein. Since this necessitated treatment in view of her clinical deterioration it was considered a myeloma-defining event.

## Conclusions

Our patient illustrates the importance of having a high index of suspicion and considering multiple myeloma as part of the differential diagnosis in patients presenting with unexplained protein-losing enteropathy due to intestinal lymphangiectasia. This case illustrates the importance of considering rare presentations of multiple myeloma in the absence of the traditional CRAB criteria. Delay in accurate diagnosis of myeloma may result in end organ damage, complications, and increased morbidity. Any end organ damage due to myeloma outside of the traditional criteria, such as in this case the ocurrence of intestinal lymphangeictasia, should also be classified as a myeloma-defining event, and should merit initiation of therapy. 
